# Low atmospheric pressure stunning as a new method for stunning broilers in Germany under ethological aspects

**DOI:** 10.1016/j.psj.2025.106224

**Published:** 2025-12-10

**Authors:** Felix Kuck, Jan Heck, Shana Bergmann, Paul Schmidt, Elke Rauch, Helen Louton, Angela Schwarzer

**Affiliations:** a Ludwig Maximilians University Munich, Munich, Germany; b Bavarian Health and Food Safety Authority, Oberschleißheim, Germany; c Statistical Consulting for Science and Research, Berlin, Germany

**Keywords:** Low atmospheric pressure stunning, Poultry, Animal welfare, Ethology

## Abstract

According to Council Regulation (EC) No 1099/2009, poultry must be stunned before slaughter or killing by using an electrical water bath or controlled atmosphere stunning. Low atmospheric pressure stunning (**LAPS**) is a novel approach to stunning poultry without process gases. The animals are placed into a sealed chamber, and the air pressure is reduced by a specific 2-phased pump curve to reach a target level of decompression. During a LAPS cycle, air oxygen decreases to under 5 % of the initial oxygen concentration and leads to unconsciousness of the animals by hypoxia. In 2018, LAPS was approved as a stunning method in the European Union (Commission Implementing Regulation [EU] 2018/723). The aim of this study was to evaluate LAPS in Germany under animal welfare aspects. On 9 trial days, 35 Ross 308 broilers were stunned using a LAPS cycle with a decompression curve in accordance with Commission Implementing Regulation (EU) 2018/723. Individual video recordings of the broilers were analyzed. The ethogram used to interpret animal behavior consisted of aversive reactions, indicators of consciousness, and indicators of unconsciousness. Additionally, microclimatic and biological factors such as age, sex, and body weight potentially influencing the LAPS cycle were recorded. The results showed that up to 43 seconds after starting the LAPS cycle, only indicators of consciousness were recorded. From 248 seconds after starting the LAPS cycle, only indicators of unconsciousness occurred. During the interval between 44 and 247 seconds in the LAPS cycle, indicators of both consciousness and unconsciousness were observed, which impaired the evaluation of whether animals were conscious or unconscious. Aversive reactions were frequently observed during this intermediate phase. Even though Commission Implementing Regulation (EU) 2018/723 lists LAPS as stunning method for slaughtering and killing broilers, further research on welfare aspects is necessary. The results of this study indicate difficulties in determining the exact time when the animals become unconscious and whether aversive reactions are still consciously perceived.

## Introduction

Germany is the fourth greatest producer of poultry meat in the European Union (**EU**). In 2024, Germany slaughtered more than 626 million broilers (Federal Statistical Office of Germany, 2025). According to Council Regulation (EC) No 1099/2009 on the protection of animals at the time of killing, animals must be stunned before slaughtering and killing. Currently, 2 common stunning methods are used in the EU: electrical water-bath stunning (**EWS**) and controlled atmosphere stunning (**CAS**). According to Council Regulation (EC) No 1099/2009, CAS is approved with different specifications for poultry, including both stunning by process gases and low atmospheric pressure stunning (**LAPS**). Commission Implementing Regulation (EU) No 2018/723 approved LAPS as a method for broiler chickens up to 4 kg in the EU for stunning, killing, and other uses. All mentioned methods have disadvantages from an animal welfare point of view. For EWS, the conscious birds are manually hung upside down by their feet in stainless steel shackles and pass an electrical water bath (Gerritzen et al., 2000; Lambooij et al., 2014). Animal welfare concerns about this procedure include stress and pain due to handling and hanging of the birds upside down in the shackles while they are conscious (Sparrey and Kettlewell, 1994; Gerritzen et al., 2000). The procedure leads to an increase in corticosterone concentration in the blood (Debut et al., 2005) and can cause bone fractures in animals with higher weight (Sparrey and Kettlewell, 1994). The electric current induces an epileptic seizure and a ventricular fibrillation by flowing through the body (Sparrey and Kettlewell, 1994). Additionally, the efficiency of EWS depends on various technical parameters, biological variations of the birds, and the number of birds simultaneously present in the water bath (Raj and O’Callaghan, 2004; Hindle et al., 2010; Lambooij et al., 2014). The most common CAS method in the EU uses carbon dioxide with multiple stages of gas concentration. First, the birds get exposed to a low concentration of less than 40 % carbon dioxide until they are unconscious. This lower concentration minimizes aversive reactions of the birds. Subsequently, the animals are exposed to an atmosphere containing 80 % to 90 % carbon dioxide. The second phase ensures that unconsciousness lasts long enough to achieve irreversible stunning (Berg and Raj, 2015). However, birds have chemoreceptors sensitive to carbon dioxide, so the gas can induce aversive reactions such as headshaking and gasping (Berg and Raj, 2015). In contrast to EWS, CAS induces unconsciousness more slowly in the birds (Berg and Raj, 2015), so the animals might be conscious for a longer period and have more negative experiences during the process. LAPS is a novel method, which is supposed to have the potential to raise animal welfare at the abattoirs by reducing handling-stress and inducing unconsciousness smoothly and irreversibly (Martin et al., 2016a). During LAPS, the animals are placed into a sealed chamber. The air pressure is reduced by a pump using a specific decompression curve to reach a target decompression point, which is held for a specific period of time until the animals are irreversibly unconscious (Cheek and Cattaruzzi, 2010). In 2017, the European Food Safety Authority (**EFSA**) published a scientific opinion on LAPS based on former research and considered LAPS to be at least equivalent in terms of animal welfare outcomes as compared with other methods listed in Council Regulation (EC) No 1099/2009 (More et al., 2017). The EFSA scientific opinion states risk factors for animal welfare, including various climate conditions and biological parameters such as genetics, age, and weight (More et al., 2017). In North America, LAPS has a “no objection” status by the US Department of Agriculture and the Canadian Food Inspection Agency (Mackie and McKeegan, 2016). It is routinely used in a poultry processing plant in Arkansas (Mackie and McKeegan, 2016; Martin et al., 2016b). During LAPS, the birds are put into the LAPS chamber without being tipped out of the crates or being shackled (Berg and Raj, 2015). According to Commission Implementing Regulation (EU) No 2018/723, in the first phase of the stunning process, the decompression rate shall not be greater than the equivalent to a reduction in pressure from standard sea level atmospheric pressure of 760Torr (101.32 kPa) to 250 Torr (33.33 kPa) for a period of at least 50 seconds. In the second phase, a minimum standard sea level atmospheric pressure of 160 Torr (21.30 kPa) shall be reached within the following 210 seconds. This procedure reduces the oxygen concentration to below 5 % of the initial concentration and brings the air pressure near the level of 15.00 kPa, where the diffusion of oxygen from the alveolus into the cell is not possible anymore (Gurung et al., 2018). According to the literature, a slow decompression like in this specific multiple-stage decompression curve is a humane method to induce hypobaric anoxia (McKeegan et al., 2013). Holloway and Pritchard (2017) reported only a minimal impact of the presence of animals when using a fully automatic LAPS machine. The manufacturer (TechnoCatch, Kosciusko, MS) had informed Holloway and Pritchard (2017) that there might be a ±10 % tolerance in reaching the pressures on time. The investigation of different decompression curves produced by different target pressures in 180.00 seconds found that at a target pressure of 26.60 kPa, broilers showed a loss of posture (**LOP**) after 34.50 seconds (Purswell et al., 2007). At a target pressure of 23.60 kPa, broilers were dead after 82.70 seconds. The estimated pressure level lethal for 99.99 % of the broilers was 19.40 kPa in 180.00 seconds (Purswell et al., 2007). Studies on the behavior of poultry during LAPS revealed similar behaviors in comparison with CAS (Vizzier-Thaxton et al., 2010; Johnson, 2014; Martin et al., 2016b), specifically headshaking, mandibulation, and open bill breathing (Mackie and McKeegan, 2016). According to Gent et al. (2020), there might be less aversive reactions caused by LAPS than by CAS. Both LOP and a motionless state are reached faster in LAPS than in CAS systems (Gent et al., 2020). Latest studies of animal behavior when CAS stunning Ross 308 broilers in 2 phases by carbon dioxide and nitrogen showed that in new and correctly executed systems, broilers showed LOP after 19.80 seconds (range 14.00 to 30.80 seconds) and motionlessness after 66.00 seconds (range 43.00 to 108.00 seconds) (Rucinque et al., 2024). The aim of the present study was to assess LAPS as a potential new stunning method for the first time on Ross 308 broilers in Germany under animal welfare aspects by using ethological parameters under laboratory conditions.

## Materials and Methods

### Procedure

This study was approved by the Bavarian government (approval number ROB-55.2-2532.Vet_02-23-28). The animal experiment was in accordance with the Directive 2010/63/EU. The experiment was conducted on 9 days from October 2023 to March 2024. During these 9 days, 25 LAPS cycles were performed under laboratory conditions at the Chair for Animal Welfare, Ethology, Animal Husbandry and Animal Hygiene, Ludwig Maximilians University of Munich, Germany. During these LAPS cycles, the animals got stunned and their behavior was observed. This study did not include a control group with CAS-stunned animals, due to the practical implementation of the 3 R rule of the Directive 2010/63/EU (reduction of animals used for animal experimentation).

### Animals

Thirty-five Ross 308 broilers (Aviagen, Huntsville, AL) were used in this study. They were raised on different commercial farms in Germany under husbandry conditions in accordance with EU Council Directive 98/58/EC, Council Directive 2007/43/EC, and the German Animal Welfare Act (2006). According to Regulation (EC) No 853/2004, Regulation (EU) 2017/625**,** Commission Delegated Regulation (EU) 2019/624, and Commission Implementing Regulation (EU) 2019/627. According to Commission all broilers underwent an antemortem inspection once, and the animal health certificate was issued by an official veterinarian Regulation (EU) 2019/627. On the day of slaughter, 4 to 6 animals for the trial day were randomly selected from the flock at an average age of 35 days and an average stock weight of 2.50 kg per animal. All selected birds were sexed, individually weighed, and visually inspected directly before the stunning procedure. Directly after the stunning process (see next section), signs of death including absence of breathing, absence of heartbeat, loss of muscle tension, eye condition, and the loss of interdigital and eye reflexes were checked by a veterinarian. Subsequently, the animals were bled out by one bleeding cut on the side of the neck according to Directive 2010/63/EU and weighed again.

### LAPS prototype and microclimatic parameters

The LAPS prototype used for this study was a custom-made prototype. The horizontal cylindric sealed chamber was made of sheet steel with a thickness of 5 mm. It was 850 mm long and had a diameter of 650 mm. The frontside door was locked by tilt locks and gasket, so it could hold the vacuum inside. The LAPS prototype was installed with 4 steel pillars on a Euro pallet made of thick plastic. A control unit (Siemens LOGO! Basic Module, Siemens, Munich, Germany) steered the air pressure controlling a pump (Edwards EDC 150, Edwards Ltd, Burgess Hill, United Kingdom) during 2 phases. To implement the 2 phases, the pump was connected to the chamber by 2 hoses, each ending at a valve. The hose for the first phase had a ball clock valve (DIN 20 ¾ inch), that for the second phase had a butterfly valve (G ½ inch). The control unit was programmed to a specific voltage output, which determined the target pressure for phases 1 and 2. It was programmed on each trial day considering the daily atmospheric air pressure, as recommended by Holloway and Pritchard (2017). The LAPS prototype was programmed to reduce the air pressure in the first phase from atmospheric pressure of the specific trial day to 33.30 kPa in at most 70.00 seconds. In the second phase, the pressure was reduced from 33.30 kPa down to 21.30 kPa in not more than 200.00 seconds. These target pressures and phase times were chosen to comply with Commission Implementing Regulation (EU) 2018/723 of 16 May 2018 amending Annexes I and II to Council Regulation (EC) No 1099/2009 on the protection of animals at the time of killing. The inside of the chamber was illuminated by 2 blue LED lamps (Paulmann E27 blue, 230 V, Paul Licht GmbH, Springe, Germany). Light intensity inside the chamber at the start of the LAPS cycle was measured once at the beginning of the experiment by a HATO ONE light meter (Hato B.V., Sittard, The Netherlands). The parameters air pressure, relative air humidity, and air temperature of the ambient atmosphere were recorded continuously in intervals of seconds starting at the beginning of the LAPS cycle until re-ventilation. Temperature and air pressure were recorded by a “PatBox” data logger (Witt-Gastechnik GmbH & Co., Witten, Germany). Relative humidity was recorded by a “NovusLogger” data logger (B+B Thermo-Technik GmbH, Donaueschingen, Germany).

### Behavior observations

The animals were never stunned alone to prevent isolation stress as an influence on the behavior as recommended by Mackie and McKeegan (2016). They were placed in a commercial poultry transport crate in the LAPS prototype. The crate (Olba B.V., Coevorden, The Netherlands) measured 77 × 58 × 42 cm (L × W × H). It was divided into 2 compartments by a Plexiglas wall, each compartment for 1 animal per LAPS cycle. Behavior was recorded using 2 GoPro Hero 7 and 2 GoPro Hero 10 cameras (GoPro Inc., San Mateo, CA) with 1 of each camera type used per bird. The ethogram used in this study comprised 32 behaviors and was based on former LAPS and CAS studies (Lambooij et al., 1999; Webster and Fletcher, 2001; Coenen et al., 2009; Mackie and McKeegan, 2016; Martin et al., 2016a, 2016b). The focus was placed on the occurrence of aversive reactions and indicators of consciousness and unconsciousness as reported in former studies on LAPS (Lambooij et al., 1999; Webster and Fletcher, 2001; Coenen et al., 2009; Mackie and McKeegan, 2016; Martin et al., 2016a, 2016b) and CAS (Santiago Rucinque et al., 2024) and the EFSA scientific opinion on LAPS (More et al., 2017), see Table 1.

**Table 1:** Ethogram of the assessed behaviors and interpretation of the behaviors, based on former investigations of low atmospheric pressure stunning (LAPS) and controlled atmosphere stunning with definition, reference, and interpretation (if no reference is given, the behavior has not been reported in previous studies on LAPS).

**Behavior**	**Definition**	**Reference for definition**	**Interpretation (Reference)**
Standing	Standing on the legs with the body fully or partly lifted off the ground	Martin et al., 2016a	Consciousness Blokhuis et al., 2004
Sitting	Legs underneath the body cavity and wings relaxed against body wall	Mackie und McKeegan, 2016	Consciousness Gerritzen et al., 2013 Verhoeven et al., 2015
Ataxia	Apparent dizziness, staggering, swaying of body and/or head, attempts to stand	Mackie und McKeegan, 2016 Martin et al., 2016a	Indicator of welfare More et al., 2017
Recovery of balance	A sudden effort to regain balance accompanied by lifting wings away from body while standing/sitting or flapping wings to try and regain balance	Webster und Fletcher, 2001	
Alertness	Restless movements of the head and/or restless movements of the body	Mackie and McKeegan, 2016	
Drowsiness	Reduced alertness, lower muscle tone, partially closed eyes or drooping eyelids		
Loss of posture	Unable to regain/maintain a controlled posture	Mackie and McKeegan, 2016 Martin et al., 2016a	Unconsciousness More et al., 2017
Flip	A convulsive movement just before complete collapse, causing the bird to fall on its back	Webster und Fletcher, 2001	
Lying	Lying once posture is lost and not perceived as purposefully controlling posture	Mackie und McKeegan, 2016 Martin et al., 2016a	Unconsciousness More et al., 2017
Motionlessness	No discernible body movements	Mackie und McKeegan, 2016 Martin et al., 2016a	Unconsciousness More et al., 2017
Convulsion	Uncontrolled twitching (visible muscular spasms within the body). Rapid/vigorous movement of the wings. A new bout was defined as following a pause of at least 1 second	Mackie und McKeegan, 2016 Martin et al., 2016a	Indicator of welfare More et al., 2017
Wing flapping	One short burst or prolonged slow/moderate movement of the wings, occurring without any twitching of the body. A new bout was defined by a pause of 1 second	Mackie und McKeegan, 2016 Martin et al., 2016a	Indicator of welfare More et al., 2017
Leg paddling	Involuntary, usually alternating, leg movements in the air or towards the ground depending on the body position of the bird. A new bout was defined by a pause of 1 second	Mackie und McKeegan, 2016 Martin et al., 2016a	
Jumping	Explosive movement from a sitting/lying position to stand up in the air and then immediately resuming sitting/lying position	Gerritzen et al., 2006 Mackie und MacKeegan, 2016 Martin et al., 2016a	Indicator of welfare More et al., 2017
Pecking movements	Moving the head back and forth in a pecking motion	Mackie und McKeegan, 2016 Martin et al., 2016a	
Normal breathing	Normal rhythmic breaths, without neck extension, open bill, or gasping	Webster und Fletcher, 2001	
Mandibulation	Repetitive and rapid opening and closing of the bill, not associated with inhalation or exhalation	Martin et al., 2016a	
Deep inhalation	Deep non-rhythmic inhalation from the mouth, may be accompanied by extension of the neck	Martin et al., 2016a	Indicator of welfare More et al., 2017
Headshaking	Rapid lateral head movement	Mackie and McKeegan, 2016 Martin et al., 2016a Gent et al., 2020	Indicator of welfare More et al., 2017
Open bill (beak) breathing	Gentle rhythmic breathing with bill (beak) open, with or without neck extension	Martin et al., 2016a	Indicator of welfare More et al., 2017
Loss of jaw tension	Bill open for more than 2 seconds without deep inhalation and/or neck extension	Mackie und McKeegan, 2016 Martin et al., 2016a	
Eyes open	Eyes fully open and focusing		Consciousness Verhoeven et al., 2015
Eyes half-open	Eyelid covers part of the eybulbus		Consciousness
Eyes closed	Eyelid fully closed, eyebulbus not visible		
Blinking	Rhythmically opening and closing the eyelid		Consciousness
Vocalizing	Any audible vocal produced by the focal bird (e.g., alarm call or peeping)	Martin et al., 2016a	Consciousness Blokhuis et al., 2004
Ruffled plumage	Animals ruffle their feathers before loss of posture		
Defecation	Voiding of feces	Webster und Fletcher, 2001	

The observation period was defined from the start of the LAPS cycle to at least the beginning of re-ventilation. The observation was performed as a focal animal sampling by continuous recording (Bateson and Martin, 2021) by reviewing the recorded video data. The recording accuracy was 1 second. The review was performed at 0.50 speed of the original video speed. The analysis that focused exclusively on the behavior of the eyes was clustered and reviewed at 0.25 speed of the original video speed. Single blinks were recorded as 1 blink in 1 second, and blinking episodes of more than 1 blink in 1 second were recorded as 1 blink in 1 second as well because the recording interval could not be set more precisely. Tonic and clonic convulsions were summarized as convulsion because it was not possible to distinguish between them.

### Statistical analysis

The microclimatic data were exported into Microsoft Excel (Version 18088, Microsoft Corporation, Redmond, WA) and synchronized with the time in seconds after the start of the LAPS cycle. The effect of relative humidity, temperature, atmospheric air pressure, body weight, and age of the animals on the time to reach the target level of decompression in phases 1 and 2 was analyzed. For this purpose, Pearson correlation coefficients were determined and the corresponding 95 % confidence intervals and *p*-values were calculated. The videos were manually analyzed by one human observer using the Microsoft Media Player App (Version 2.0, Microsoft Corporation, Redmond, WA). The results were also transferred into Microsoft Excel (Version 18088, Microsoft Corporation, Redmond, WA) and synchronized to the data of LAPS cycles and microclimatic data. Intra- and inter-observer reliability was tested on 2 animals, 1 in the beginning of the study and 1 at the end of the study, for the behaviors ataxia, convulsion, deep inhalation, wing flapping, jumping, open bill breathing, headshaking, LOP, and motionlessness. A prevalence-adjusted and bias-adjusted kappa value was calculated for each second of the videos used for the inter- and intra-observer reliability tests. For each animal, it was recorded how often the investigated behaviors were exhibited. Poisson models were then used to determine whether the physiological parameters body weight, age, and sex affected the mean count of these behaviors. For continuous parameters, results of this analysis are presented as the multiplicative change in the expected count (i.e., the incidence rate ratio) for a 1-unit increase in the predictor, together with their 95 % confidence intervals and 2-sided *p*-values, all reported on the original count scale. For sex, results present the multiplicative change in expected count when considering female instead of male animals. Behavioral durations were recorded for each animal in seconds; because a behavior could occur repeatedly, multiple durations per individual were available. The influence of physiological parameters on these durations was examined with linear mixed-effects models, specifying body weight and age (continuous) and sex (categorical) as fixed effects and animal ID as a random effect. Results are reported as the change in expected duration for a 1-unit increase in each continuous predictor, accompanied by 95 % confidence intervals and 2-sided *p*-values. For sex, the results represent the difference in expected duration for females relative to males. All statistical analyses were made using R programming language (R Core Team, 2024).

## Results

### Animals

The live body weight of the 35 broilers was on average 2,040 g (SD 203 g) with a range of 1,642 to 2,615 g. The body weight after bleeding postmortem was on average 1,984 g (SD 200 g) with a range of 1,582 to 2,551 g. The sex distribution was 34 % male (12 broilers) and 66 % female (23 broilers). The average age was 35 days (SD 0.84 days) with a range of 34 to 37 days.

### LAPS cycle

**Fig. 1:**
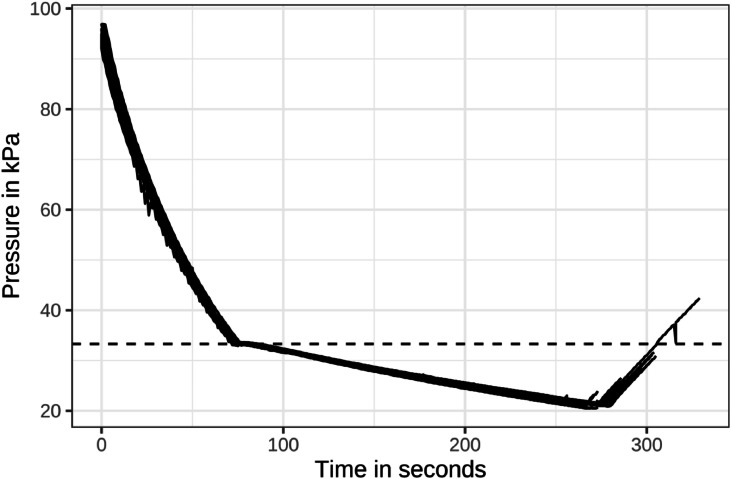
All low atmospheric pressure stunning cycles of the study superimposed (dashed line = 33.3 kPa marker).

Light intensity inside the chamber at the start of the LAPS cycle was 26 lx. The duration of a LAPS cycle was on average 270 seconds with a range of 252 to 278 seconds. The target pressure of phase 1 (33.30 kPa) was reached on average after 83 seconds (73 to 86 seconds), that of phase 2 (21.30 kPa) on average after 270 seconds (252 to 278 seconds) after the start of the LAPS cycle. Re-ventilation of the chamber after decompression always took 299 seconds. As the reduction in oxygen is proportional to the reduction in pressure (Holloway and Pritchard, 2017), an oxygen level below 5 % was reached because the pressure was reduced on average by 77.89 % from starting pressure. This was in accordance with Commission Implementing Regulation (EU) 2018/723. Fig. 1 shows all LAPS cycles of the study superimposed. Both LAPS phases therefore met the requirements of Council Regulation (EC) No 1099/2009, albeit with some variance (Table 2).

**Table 2:** Descriptive analysis of the duration of cycle phases 1 and 2 during low atmospheric pressure stunning, showing the time in seconds until the target pressure of phase 1 (33.30 kPa) or phase 2 (21.30 kPa) was reached.

**Statistic**	**Time (seconds) to reach target pressure**	
	**Phase 1**	**Phase 2**
Mean (SD)	81.10 (3.90)	268.40 (5.50)
Median (IQR)	83.00 (80.00, 84.00)	270.00 (268.00, 271.00)
Range	73.00–86.00	252.00–278.00

IQR = interquartile range.

To explain this variance, microclimatic ambient and physiological parameters of the animals were considered regarding their influence on the LAPS decompression curves. Table 3 summarizes the descriptive analysis of the microclimatic parameters relative humidity, temperature, and atmospheric air pressure and the physiological parameters live body weight, body weight after bleeding, and age. For phase 2, we found statistically significant effects. The initial atmospheric air pressure had a positive effect (higher initial pressure led to a longer time to reach the target pressure), whereas the 2 weight characteristics had a negative effect (a higher body weight led to a shorter time to reach the target pressure) (Table 4). There was an increase in pressure at the switch between phase 1 and phase 2 of the LAPS cycle for an average 0.136 kPa (range 0.1 kPa to 0.3 kPa) in 4.60 seconds (range 4.00 to 9.00 seconds). The duration of pressure increase was below the 10 % tolerance range for pressure and time.

**Table 3:** Descriptive analysis of microclimatic parameters inside the chamber for low atmospheric pressure stunning and of physiological parameters of the broilers.

**Statistic**	**Parameter**					
	**Relative humidity (%)**	**Atmospheric air pressure (kPa)**	**Temperature (°C)**	**Live body weight (g)**	**Body weight after bleeding (g)**	**Age (days)**
Mean (SD)	42.40 (8.70)	94.60 (1.50)	22.30 (0.88)	2,040.00 (203.00)	1,984.00 (200.0)	35.00 (0.84)
Median (IQR)	44.20 (40.10, 47.00)	9.48 (94.10, 95.60)	22.00 (22.00, 23.00)	2,051.00 (1,922.00, 2,157.00)	1,987.00 (1,861.00, 2,100.00)	35.00 (34.00, 35.00)
Range	0.00–53.50	89.900–96.900	20.50–24.00	1,642.00–2,615.00	1,582.00–2,551.00	34.00–37.00

IQR = interquartile range.

**Table 4:** Influence of microclimatic parameters and of physiological parameters of the broilers on the duration of cycle phases 1 and 2 during low atmospheric pressure stunning, with the phases defined as the time in seconds until the target pressure of phase 1 (33.30 kPa) or phase 2 (21.30 kPa) was reached and with bold letters indicating statistical significance (N = 26 broilers).

**Parameter**	**Phase 1**	**Phase 2**
	**Correlation**^1^ [**95% CI**] ***p*-value**	**Correlation**^1^ [**95% CI**] ***p*-value**
Relative humidity (%)	0.33 [−0.01, 0.61] *p* = 0.057	−0.15 [−0.46, 0.20] *p* = 0.407
Atmospheric air pressure (kPa)	0.022 [−0.013, 0.053] *p* = 0.210	**0.056 [0.027, 0.075**] ***p*** < **0.001**
Temperature (°C)	**−0.54 [−0.75, −0.24**] ***p*** = **0.001**	−0.12 [−0.44, 0.30] *p* = 0.837
Live body weight (g)	−0.20 [−0.15, 0.51] *p* = 0.265	−**0.46** [**−0.69, −0.15**] ***p*** = **0.006**
Body weight after bleeding (g)	0.22 [−0.13, 0.52] *p* = 0.218	**−0.46 [−0.69, −0.15**] ***p*** = **0.006**
Age (days)	**0.43 [0.10, 0.67]** ***p*** = **0.013**	−0.32 [−0.59, 0.02] *p* = 0.064

CI = confidence interval.

1 Pearson correlation coefficient.

### Behavior observations

The inter-observer reliability was 96 % and the intra-observer reliability 98 %. Table 5 summarizes the descriptive analysis of the behaviors of the broilers during the LAPS cycle. No broiler was at any time fully out of view; however, not all body parts could be assessed all the time during the LAPS cycle. Therefore, the number of broilers for the calculation of proportion in Table 5 varied throughout the duration of the stunning process. During the LAPS cycle, 100 % of the broilers showed LOP and lying, but only 80.0 % of the broilers reached the point of motionlessness before re-ventilation started. Wing flapping was performed by 33 broilers (94.3 %), and all broilers showed convulsions. On average, the broilers exhibited 6.46 convulsions (SD 3.10), with an average duration of 4.60 seconds (SD 5.21 seconds). The maximum recorded on 1 broiler was 16 convulsions, with the last convulsion observed 273 seconds after the start of the LAPS cycle. Jumping was observed in 9 broilers (25.7 %), 1 broiler jumped twice and all others once. Eyes open occurred from 0 to 62 seconds and was shown 5.86 times (SD 2.92), eyes half-open occurred from 9 to 198 seconds and was shown 4.54 times (SD 2.68), and eyes closed occurred from 74 to 174 seconds and was shown 5.46 times (SD 2.51).

**Table 5:** Descriptive analysis of the behavior parameters during the low atmospheric pressure stunning (LAPS) cycle, showing the frequencies and durations of all behaviors and the time during the LAPS cycle (seconds after start) at which the behavior was observed for the first and for the last time (because not all behaviors were observed in all broilers, the number of broilers expressing a behavior and the percentage of the total number of broilers [n = 35] are given).

**Behavior**	**Number of broilers (%)**	**Frequency (events)** **Mean (SD)** **Range** **95% CI**	**Duration (seconds)** **Mean (SD)** **Range** **95% CI**	**First appearance (seconds after start)** **Mean (SD)** **Range** **95% CI**	**Last appearance (seconds after start)** **Mean (SD)** **Range** **95% CI**
Standing	9 (25.7)	0.46 (0.89) 0.00–3.00 [0.28, 0.75]	19.83 (22.16) 1.00–55.00 [2.80, 36.87]	23.78 (30.04) 0.00–78.00 [0.68, 46.87]	45.78 (28.70) 0.00–78.00 [23.72, 67.84]
Sitting	35 (100)	1.26 (0.66) 1.00–4.00 [0.94, 1.69]	62.77 (27.89) 1.00–98.00 [53.19, 72.35]	6.63 (18.38) 0.00–60.00 [0.31, 12.94]	13.03 (24.59) 0.00–75.00 [4.58, 21.48]
Ataxia	34 (97.1)	1.06 (0.34) 0.00–2.00 [0.77, 1.46]	20.63 (9.75) 4.00–53.00 [17.23, 24.03]	60.32 (9.41) 34.00–76.00 [57.04, 63.61]	62.03 (12.02) 34.00–98.00 [57.84, 66.22]
Recovery of balance	30 (85.7)	1.94 (1.33) 0.00–4.00 [1.53, 2.46]	1.33 (0.60) 1.00–4.00 [1.11, 1.56]	76.03 (12.12) 59.00–103.00 [71.51, 80.56]	83.23 (13.55) 59.00–110.00 [78.17, 88.29]
Alertness	11 (31.4)	0.37 (0.65) 0.00–3.00 [0.22, 0.64]	21.39 (17.27) 1.00–63.00 [9.79, 33.00]	10.18 (19.32) 0.00–50.00 [0.00, 23.16]	17.36 (27.92) 0.00–79.00 [0.00, 36.12]
Drowsiness	31 (88.6)	2.31 (1.76) 0.00–7.00 [1.86, 2.88]	11.60 (7.28) 2.00–30.00 [8.93, 14.27]	89.06 (15.06) 72.00–132.00 [83.54, 94.59]	135.61 (50.98) 74.00–258.00 [116.91, 154.31]
Loss of posture	35 (100)	1.00 (0.00) 1.00–1.00 [0.72, 1.39]	198.77 (8.72) 173.00–217.00 [195.78, 201.77]	75.09 (8.36) 59.00–98.00 [72.21, 77.96]	75.09 (8.36) 59.00–98.00 [72.21, 77.96]
Flip	16 (45.7)	0.49 (0.56) 0.00–2.00 [0.30, 0.78]	1.19 (0.40) 1.00–2.00 [0.97, 1.40]	164.44 (46.57) 89.00–274.00 [139.62, 189.25]	166.69 (47.10) 89.00–274.00 [141.59, 191.79]
Lying	35 (100)	1.09 (0.28) 1.00–2.00 [0.79, 1.49]	189.84 (28.81) 98.00–217.00 [179.95, 199.74]	75.09 (8.36) 59.00–98.00 [72.21, 77.96]	76.23 (8.35) 59.00–98.00 [73.36, 79.10]
Motionlessness	28 (80.0)	0.80 (0.41) 0.00–1.00 [0.55, 1.16]	46.04 (26.54) 2.00–97.00 [35.74, 56.33]	228.29 (27.02) 176.00–276.00 [217.81, 238.76]	228.29 (27.02) 176.00–276.00 [217.81, 238.76]
Convulsion	35 (100)	6.46 (3.10) 2.00–16.00 [5.67, 7.36]	4.60 (5.21) 1.75–33.50 [2.81, 6.39]	134.31 (38.38) 70.00–227.00 [121.13, 147.50]	215.83 (35.11) 147.00–273.00 [203.77, 227.89]
Wing flapping	33 (94.3)	2.29 (1.27) 0.00–4.00 [1.84, 2.85]	2.23 (1.63) 1.00–9.00 [1.65, 2.81]	86.03 (16.73) 64.00–156.00 [80.10, 91.96]	113.00 (33.28) 72.00–200.00 [101.20, 124.80]
Leg paddling	35 (100)	7.71 (3.30) 2.00–15.00 [6.85, 8.69]	2.28 (1.03) 1.00–5.60 [1.93, 2.63]	95.09 (25.90) 67.00–184.00 [86.19, 103.98]	208.69 (37.61) 114.00–278.00 [195.76, 221.61]
Jumping	9 (25.7)	0.29 (0.52) 0.00–2.00 [0.15, 0.53]	1.00 (0.00) 1.00–1.00 [1.00, 1.00]	135.67 (79.57) 71.00–260.00 [74.50, 196.83]	149.33 (78.80) 71.00–260.00 [88.76, 209.90]
Pecking movements	5 (14.3)	0.29 (0.83) 0.00–4.00 [0.15, 0.53]	1.05 (0.11) 1.00–1.25 [0.91, 1.19]	58.60 (28.22) 33.00–105.00 [23.56, 93.64]	65.6 (22.84) 49.00–105.00 [37.24, 93.96]
Normal breathing	35 (100)	3.46 (2.31) 1.00–13.00 [2.89, 4.13]	72.79 (50.01) 8.77–206.00 [55.62, 89.97]	0.97 (5.75) 0.00–34.00 [0.00, 2.95]	122.74 (66.62) 0.00–265.00 [99.86, 145.63]
Mandibulation	2 (5.7)	0.29 (1.53) 0.00–9.00 [0.15, 0.53]	4.00 (2.83) 2.00–6.00 [−21.41, 29.41]	60.50 (85.56) 0.00–121.00 [0.00, 829.23]	76.00 (63.64) 31.00–121.00 [0.00, 647.78]
Deep inhalation	30 (85.7)	4.11 (3.50) 0.00–16.00 [3.49, 4.84]	1.25 (0.59) 1.00–3.60 [1.03; 1.47]	153.4 (76.15) 8.00–259.00 [124.96, 181.84]	213.70 (29.91) 158.00–270.00 [202.53, 224.87]
Headshaking	29 (82.9)	2.29 (1.71) 0.00–5.00 [1.84, 2.85]	1.81 (1.96) 1.00–11.00 [1.07, 2.56]	62.66 (18.02) 10.00–93.00 [55.80, 69.51]	85.21 (36.11) 43.00–213.00 [71.47, 98.94]
Open bill breathing	2 (5.7)	0.29 (1.53) 0.00–9.00 [0.15, 0.53]	4.00 (2.83) 2.00–6.00 [−21.41, 29.41]	60.50 (85.56) 0.00–121.00 [0.00, 829.23]	76.00 (63.64) 31.00–121.00 [0.00, 647.78]
Loss of jaw tension	30 (85.7)	3.4 (2.58) 0.00–11.00 [2.84, 4.07]	9.33 (7.13) 1.00–30.00 [6.67, 11.99]	89.67 (28.05) 52.00–151.00 [79.19, 100.14]	168.77 (56.35) 52.00–258.00 [147.72, 189.81]
Eyes open	35 (100)	5.86 (2.92) 1.00–12.00 [5.11, 6.72]	24.16 (17.42) 8.50–81.00 [18.18, 30.15]	1.86 (10.47) 0.00–62.00 [0.00, 5.45]	178.34 (72.33) 0.00–264.00 [153.50, 203.19]
Eyes half-open	33 (94.3)	4.54 (2.68) 0.00–12.00 [3.89, 5.31]	6.55 (5.87) 1.00–27.33 [4.46, 8.63]	93.03 (30.42) 9.00–198.00 [82.24, 103.82]	206.48 (45.46) 79.00–271.00 [190.37, 222.60]
Eyes closed	35 (100)	5.46 (2.51) 2.00–14.00 [4.74, 6.29]	11.87 (6.99) 1.33–26.25 [9.47, 14.27]	99.51 (20.12) 74.00–174.00 [92.60, 106.43]	205.37 (48.90) 97.00–276.00 [188.58, 222.17]
Blinking	35 (100)	20.37 (5.94) 6.00–32.00 [18.93, 21.92]	1.54 (0.50) 1.00–3.64 [1.37, 1.72]	6.80 (11.46) 0.00–69.00 [2.86, 10.74]	126.66 (61.89) 57.00–245.00 [105.40, 147.92]
Vocalizing	6 (17.1)	0.43 (1.14) 0.00–4.00 [0.26, 0.71]	1.88 (1.19) 1.00–3.75 [0.63, 3.12]	154.00 (72.92) 80.00–254.00 [77.48, 230.52]	161.33 (74.59) 81.00–266.00 [83.05, 239.61]
Ruffled plumage	23 (65.7)	0.8 (0.72) 0.00–3.00 [0.55, 1.16]	25.91 (17.03) 5.00–79.00 [18.55, 33.28]	67.39 (12.91) 42.00–94.00 [61.81, 72.97]	78.91 (30.72) 42.00–174.00 [65.63, 92.20]
Defecation	17 (48.6)	0.54 (0.61) 0.00–2.00 [0.35, 0.85]	1.06 (0.24) 1.00–2.00 [0.93, 1.18]	55.65 (13.83) 30.00–79.00 [48.53, 62.76]	65.82 (29.05) 30.00–158.00 [50.89, 80.76]

CI = confidence interval; IQR = interquartile range.

**Fig. 2:**
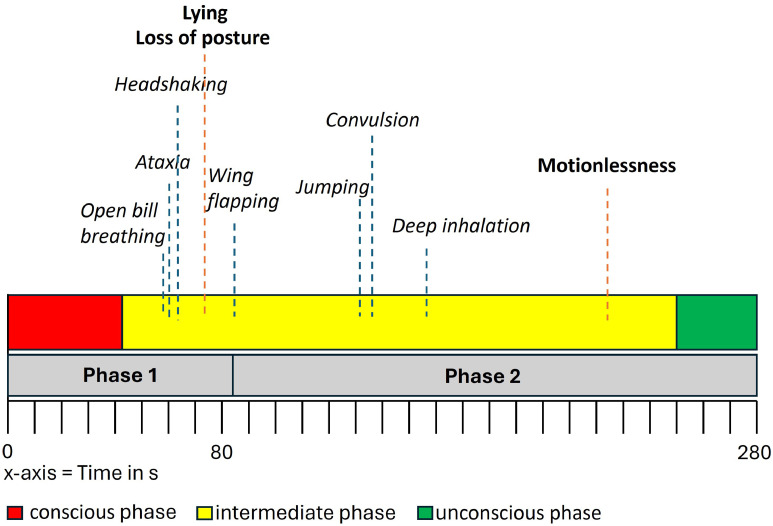
Timeline of the low atmospheric pressure stunning (LAPS) cycles. Behaviors interpreted as indicators of unconsciousness (in bold letters) and behaviors interpreted as indicators of welfare (in italic letters) are marked in their mean time of appearance; gray bars = LAPS phases; colored bars = states of consciousness; red bar = conscious phase; yellow bar = intermediate phase; green bar = unconscious phase.

Physiological parameters had an effect on the number of events (Supplementary Material, Table S1) and the duration of some of the investigated behaviors (Supplementary Material, Table S2). Heavier broilers (live body weight and body weight after bleeding) showed more loss of jaw tension (*p* = 0.015 and *p* = 0.020), vigilance (*p* < 0.015 and *p* < 0.015), and normal breathing (*p* = 0.027 and *p* = 0.032) than lighter broilers, as shown by Poisson model analysis. Male broilers showed higher frequencies of convulsions (*p* = 0.038), open bill breathing (*p* = 0.005), loss of jaw tension (*p* < 0.001), and vigilance (*p* = 0.038), whereas female broilers showed more events of vocalizing (*p* = 0.004), pecking movements (*p* = 0.002), drowsiness (*p* = 0.026), and standing (*p* < 0.001). Physiological characteristics had an effect not only on the frequency but also on the duration of certain behaviors. The duration of LOP (*p* = 0.004) and open bill breathing (*p* = 0.009) was longer in female than in male broilers, whereas the duration of sitting (*p* = 0.025) and normal breathing (*p* = 0.012) was longer in male than in female broilers. Age had a significant negative effect (*p* = 0.035) on the duration of LOP, with older broilers showing LOP later than younger ones. Older broilers showed more vigilance (*p* = 0.222), fewer events of deep inhalation (*p* = 0.016), and a shorter period for LOP (*p* = 0.035) than younger broilers. The behaviors interpreted as conscious and those interpreted as unconscious are summarized in Table 6 as indicators of consciousness and indicators of unconsciousness as they occurred during LAPS. The average time broilers showed indicators of consciousness alone was until 43 seconds (SD 19 seconds), with a range of 3 to 77 seconds, after the start of the LAPS cycle. Indicators of unconsciousness occurred alone on average 248 seconds (SD 44.00 seconds), with a range of 159 to 329 seconds, after the start of the LAPS cycle. The LOP occurred in 100 % of the broilers and the time of first occurrence was on average after 75.09 seconds (range 59 to 98 seconds), and motionlessness occurred for the first time on average after 228.29 seconds (range 176 to 276 seconds). Thus, the unconscious phase began earliest at 159 seconds and latest at 329 seconds. The intermediate phase is the phase from the end of the conscious phase until the beginning of the unconscious phase, when indicators of consciousness and indicators of unconsciousness were observed at the same time. This intermediate phase lasted on average from 43 seconds (SD 19 seconds) after start to 248 seconds (SD 44 seconds) after start, so the intermediate phase could last up to 205 seconds. The maximum interval of the intermediate phase was 217 seconds between the time when only indicators of consciousness in the conscious phase and only indicators of unconsciousness in the unconscious phase occurred. Fig. 2 shows an overview of the conscious, intermediate, and unconscious phases with the mean time until occurrence of the indicators of welfare and the indicators of unconsciousness. The longest time for reaching LOP was for 1 broiler 98 seconds after start, and motionlessness was reached by 1 broiler after 276 seconds, 4 seconds before the period of re-ventilation in LAPS.

**Table 6:** Descriptive analysis of elapsed time and decompression values during the low atmospheric pressure stunning cycles, considering the occurrence of only indicators of consciousness or only indicators of unconsciousness (N = 35 broilers).

**Statistic**	**Time or pressure until which only indicators of consciousness occurred**	**Time or pressure from which on only indicators of unconsciousness occurred**
	**Time (seconds)**	
Mean (SD)	43.00 (19.00)	248.00 (44.00)
Median (IQR)	48.00 (36.00, 54.00)	262.00 (213.00, 283.00)
Range	3.00–77.00	159.00–329.00
	**Pressure (kPa)**	
Mean (SD)	52.80 (15.50)	25.40 (4.10)
Median (IQR)	46.90 (44.00, 55.70)	24.50 (23.00, 26.30)
Range	33.60–91.00	21.10–42.40

IQR = interquartile range.

## Discussion

The performed LAPS cycle was in accordance with Council Regulation (EC) No 1099/2009. The LAPS cycle was affected by the parameters air pressure and body weight of the broilers. For phase 2, we found significant effects. A higher initial atmospheric air pressure led to a longer time to reach the target pressure of phase 2, and a higher body weight led to a shorter time to reach the target pressure of phase 2. When behavioral parameters of consciousness and unconsciousness were assessed, the point of unconsciousness was difficult to determine for Ross 308 broilers. There was an intermediate phase in which indicators of consciousness and indicators of unconsciousness occurred at the same, impeding the assessment of the state of consciousness. LAPS did not induce unconsciousness fast and directly. The occurrence of indictors of welfare in the long intermediate phase suggests that animals might suffer during the stunning process.

### LAPS cycle

The duration of LAPS cycles in this study was similar to the cycles described in studies on commercially used units (Vizzier-Thaxton et al., 2010; McKeegan et al., 2013). The durations of phases 1 and 2, an oxygen level of less than 5 %, and the target pressures were in accordance with Commission Implementing Regulation (EU) 2018/723. Even though every LAPS decompression cycle used in our study complied with all target duration times and target pressures named in the Regulation (EU) 2018/723, the decompression rate per second for both phases is undefined by the Regulation (EU) 2018/723. The Regulation (EU) 2018/723 requires a gradual decompression, which was adhered to in our study. The recommended target level of decompression of 26.60 kPa to stun broilers in accordance with animal welfare (Purswell et al., 2007) was reached in every LAPS cycle in the present study. The data of microclimatic conditions within the LAPS prototype showed correlation with the parameters air temperature and relative humidity of the ambient atmosphere, as also found in a previous study (Holloway and Pritchard, 2017). However, our study was conducted indoors, resulting in very little temperature variation between cycles, and therefore an influence of a wider range of temperatures cannot be excluded. However, higher ambient air pressure at the start of a LAPS cycle extended the time to reach the target pressure of phase 2. An older age of the broilers was correlated with a reduction in time to reach the target pressure of phase 1, and a higher body weight (before and after loss of blood) was correlated with a reduction in time to reach the target pressure of both phases. This was likely due to the positive correlation of body weight and age in animals of the same genetic under similar husbandry conditions. Higher body weights are associated with larger body volumes (Blocksiepen, 2016), and thus the volume of air in the LAPS chamber might be less owing to the higher body volumes of heavier broilers. The reason why the body volume had no effect on the decompression time in phase 1 but did so in phase 2 might be that in phase 1 the decompression rate is much faster than in phase 2. Therefore, the effect in phase 1 might have been too short to measure it. The increase in pressure at the switch between phase 1 and phase 2 of the LAPS cycle could have been caused by a backflow of air from the hoses leading to the valves and into the chamber or by the air expanse in the moment of a short (2.00 seconds) break of pump-down. Even a fully automatic LAPS device has a 10 % tolerance when switching from phase 1 to phase 2. This means that the time and air pressure can deviate by up to 10 % from the programmed cycle. The prototype in this study showed an average deviation of only 4.60 seconds, which was within the tolerance range of the commercially available LAPS device (Holloway and Pritchard, 2017). Because the LAPS device used in this study contained 2 valves, the comparability with other studies using a 1-valve LAPS device (Holloway and Pritchard, 2017; Gent et al., 2020) and commercially available LAPS devices (Mackie and McKeegan, 2016) might be limited. On the other hand, the LAPS prototype reached the target pressures required by the Commission Implementing Regulation (EU) 2018/723. Owing to the seasonal changes in weather between a middle European fall, winter and spring, which might have an impact on the LAPS cycle, an outdoor positioning of the LAPS device needs to be further investigated.

### Behavior observations

The LAPS cycle can be divided into 3 phases: i) pre-stunning phase (preparation of animals for stunning), ii) stunning phase, subdivided into an induction phase (the period from the start of the intervention until the onset of loss of consciousness) and an unconsciousness phase (the period between the onset of loss of consciousness until the killing intervention), and iii) death onset phase (the period from the start of the killing intervention until onset of death) (More et al., 2017). Hence, behaviors before LOP are considered aversive and relevant for animal welfare, and behaviors after LOP are considered a to be a product of seizures (jump, convulsions). Motionless in the behavioral indicator that suggest brain dead (More et al., 2017). The simultaneous occurrence of indicators of consciousness and indicators of unconsciousness in this study complicated the assessment of whether a broiler was conscious or not during the process, so there was an intermediate phase in the stunning phase. Latest studies of animal behavior during CAS of 39 days old Ross 308 broilers in 2 phases by carbon dioxide and nitrogen showed that in new and correctly executed systems, broilers showed LOP after a shorter period in both systems (Rucinque et al., 2024), than in our LAPS trial. However, age and weight of the broilers used by Rucinque et al., (2024) were different to the ones used in our LAPS-study. Therefore, we cannot our results with CAS-stunning as our study did not include a CAS-stunned reference group. Coenen et al. (2009) concluded that during CAS, the time until onset of unconsciousness in processes using only carbon dioxide is 50 seconds whereas in processes using carbon dioxide and nitrogen, it is 33 seconds. In this study, it was not possible to determine clearly the time of onset of unconsciousness because of the long intermediate phase, during which the broilers showed indicators of consciousness and indicators of unconsciousness at the same time. The faster loss of consciousness during CAS is created by a fall of the blood pH induced by the acid trait of carbon dioxide (Battula et al., 2008). This effect is absent in LAPS because of the absence of this process gas. In addition, LOP during LAPS of Ross 708 broilers occurred after 80.70 seconds (Mackie and McKeegan, 2016) and of Ross 308 broiler breeders after 86.60 seconds (Gent et al., 2020). Cobb broilers showed LOP after 54.70 seconds in dark LAPS, after 55.90 seconds in illuminated LAPS (Martin et al., 2016b), and in the quickest case after 40.70 seconds (More et al., 2017). Thus, Ross breeds showed the longest time to LOP of all tested broiler breeds in former LAPS trials. This could indicate an influence of broiler genetics on the occurrence of LOP. Therefore, Ross genetics might have an influence on the onset of unconsciousness. This hypothesis is supported by the long occurrence of indicators of consciousness and the late onset of indicators of unconsciousness alone, as found in this study. The EFSA scientific opinion on LAPS reported that birds were unconscious after an average 70.00 seconds (More et al., 2017), whereas in our study indicators of consciousness still occurred after 70.00 seconds and the broilers were interpreted to enter an intermediate phase. The light inside the LAPS prototype in our study was blue, which is perceived differently by poultry (Lewis and Morris, 2000) and is commonly used to calm broilers down (Gussem et al., 2013). It has been shown that the illumination of the chamber has no impact on the behavior of broilers during LAPS (Martin et al., 2016b), so light might not have an impact on LOP whereas genetics might do. In addition, body weight and age of the Ross 308 broilers used in this study had an influence on the assessed behaviors. Higher weight led to more normal breathing and an older age led to fewer events of deep inhalation, which can be interpreted as a quicker onset of hypoxia in these broilers with less respiratory disruption. Thus, age and body weight had similar effects, which is not surprising because body weight increases with increasing age of the birds. Importantly, older broilers showed more vigilance and a shorter period of LOP, so this might mean they became unconscious later than younger broilers and experienced hypoxia while being conscious. Male broilers showed more convulsions, which could be interpreted as more suffering than experienced by female broilers. Summarized, this study supports the key parameters of microclimatic conditions defined in Commission Implementing Regulation (EU) 2018/723 and the key biological parameters defined by the EFSA as having an impact on animal welfare. The indicators of (impaired) animal welfare were observed while the broilers were conscious. Our finding that aversive behaviors occurred while the broilers were conscious is highly relevant for the assessment of LAPS under animal welfare aspects. We found more broilers showing some of the aversive behaviors (ataxia, wing flapping, deep inhalation, convulsion) during LAPS compared to other studies examining CAS (Santiago Rucinque et al., 2024). Ataxia, headshaking, and jumping are behaviors frequently reported in studies on the behavior of broilers during stunning with LAPS (More et al., 2017) and CAS (Gerritzen et al., 2004), and these behaviors occur before the onset of LOP. Open bill breathing and deep inhalation are associated with hypoxia and occur during stunning with both CAS and LAPS (Martin et al., 2016b). Gent et al. (2020) interpreted wing flapping as an aversive reaction if the bird was conscious during the stunning process, whereas Vizzier-Thaxton et al. (2010) interpreted wing flapping in the first 60.00 seconds of LAPS as a possible indicator of the changing atmosphere at the start of decompression and thus as not aversive. Wing flapping and convulsion have been discussed to be associated with hypoxia (Berg and Raj, 2015; Gent et al., 2020) and a consequence of a myoclonic seizure. If they are, the occurrence of these behaviors must be seen critically because it might mean that the animals experienced hypoxia at full consciousness. We observed wing flapping while the broilers were conscious, and after the onset of unconsciousness we interpreted a wing flap as convulsion (both tonic and clonic). Altogether, the number of broilers showing aversive behaviors while they were conscious was higher than in the latest CAS systems (Santiago Rucinque et al., 2024). Before the onset of LOP, some of the boilers showed a ruffled plumage, which is a sign of disturbed general well-being observed in birds (Kaleta et al., 2011) and specifically in broilers is seen as a sign of potential sickness and disturbed general well-being (Gussem et al., 2013). The expansion of air in the body could cause distress or pain for the animals during LAPS. Mackie and McKeegan (2016) and Martin et al. (2020) could not rule out the possibility of distress or pain caused by expanding air in the intestines of the animals during LAPS. Humans undergoing decompression have reported that they were feeling pain in the abdomen and a prickling, creeping feeling in the hands until the hands were numb (Haymaker and Johnston, 1955). Some of the broilers in this study showed headshaking while they had a ruffled plumage. Headshaking has been interpreted as aversive reaction to gas during CAS, but it also occurs during LAPS (Mackie and McKeegan, 2016). It can be a sign of hypoxic distress, so it must be seen as indicator of distress and suffering (More et al., 2017). Mackie and McKeegan (2016) interpreted headshaking as an indicator of disorientation, discomfort, and respiratory distress while undergoing LAPS. In conclusion, these 2 indicators (headshaking and ruffled plumage) occurring together must at least be considered as evidence of disturbance of the well-being of broilers caused by distress or at least discomfort during LAPS. Furthermore, as far as we know, our study is the first in which vocalizing was observed during LAPS, another finding supporting our conclusion. During the stunning process, we observed periods of drowsiness in the beginning with following awakening, as described in the EFSA scientific opinion (More et al., 2017). The ethograms of former LAPS studies did not include behavioral observation of the eye. The present study is the first study that assesses, describes, and interprets the state of the eye opening and blinking in broilers during a LAPS cycle. There were single blinks and blinking episodes of more than 1 blink in 1 second, so the interval of 1 second might not be accurate enough for blinking. Even though the recording of blinking was difficult, the observation technique only had an impact on the number of events and not on their durations. Eyes open, eyes half-open, and blinking occurred in the state of drowsiness and the intermediate phase until they disappeared at the beginning of the unconscious phase. In the state of drowsiness, the broilers woke up several times. Flight pilots undergoing slow decompression have reported that they experienced loss of motoric skills and loss of consciousness without waking up (as cited in Mackie and McKeegan, 2016). We assume there must be causes either waking the broilers up, for example discomfort or pain caused by the decompression, or preventing them from reaching a deep state of unconsciousness in the first place. The first indicator of unconsciousness that appeared in most broilers was LOP, but indicators of consciousness, for example eyes open and headshaking, still occurred at the same time. The last indicator of unconsciousness that appeared was the onset of motionlessness. Because all broilers had their eyes open and were blinking at least at one point during the intermediate phase, we cannot exclude that the broilers might have been conscious while already showing LOP. According to the EFSA scientific opinion, the period starting from 51.00 seconds onwards for 20.00 seconds is the most important period for animal welfare (More et al., 2017). During this period, the animals are potentially conscious and show aversive reactions. Former studies were mainly focused on the investigation of the first appearance of indicators of unconsciousness and did not interpret the disappearance of indicators of consciousness (Purswell et al., 2007; Mackie and McKeegan, 2016; Martin et al., 2016b, 2016c). The last indicator of consciousness in our study disappeared after 248.00 seconds, but the indicators of consciousness “sitting” and “standing” disappeared earlier, after 75.00 and 78.00 seconds, respectively. Until this time, it is certain that the birds were still conscious. We therefore conclude that they also experienced aversive behaviors being conscious. Furthermore, the onset of LOP is only a sign of unconsciousness if it is a LOP without attempts of recovery of balance afterwards (Gerritzen et al., 2004). However, it cannot be excluded, that decompression could induce some pain and can activate some nociceptors which might end up looking like “recovery of balance”. In our investigation, broilers at the onset of LOP still showed signs of consciousness, and recovery of balance occurred the last time at a maximum of 110.00 seconds. Council Regulation (EC) No 1099/2009 defines LOP not to be the same as being unconscious or stunned. Instead, it defines clear parameters for the state of unconsciousness and the state of insentience. Council Regulation (EC) No 1099/2009 states that the state of consciousness in animals is the ability to feel emotions and control movements voluntarily and defines sensitivity as the ability to feel pain. At the same time, it names stunning as a process that causes loss of consciousness and sensibility without pain. An animal is fully stunned when it does not show any reflexes or reactions to stimuli such as sound, odor, light, or physical contact. An animal that shows LOP and tries to recover its balance cannot be interpreted as unconscious in accordance with Council Regulation (EC) No 1099/2009. To assess a stunning method under the implications of animal welfare poses high demands because not only the stunning process itself is decisive to assess the suitability of the method. The pre-stunning phase is as important as the process itself. A stunning method is acceptable if it results in minimal signs of agitation and distress while an animal is conscious, and it must guarantee the onset of unconsciousness for every animal as fast as possible (Coenen et al., 2009). If the loss of consciousness happens smoothly and rapidly, it reduces the distress in birds to a minimum level (Gerritzen et al., 2000). LAPS can possibly improve the process before stunning from an animal welfare point of view because the broilers do not get tipped out of the crates and handling and shackling while being conscious is not necessary, which leads to a lower level of corticosterone (Vizzier-Thaxton et al., 2010). In addition, LAPS does not use process gas whereas CAS methods use greenhouse gases, and thereby LAPS is more ecological and safer for the employees at the abattoir (Vizzier-Thaxton et al., 2010). Future studies should focus on the adaptation and improvement of the LAPS decompression curve, preferably using a fully automatic LAPS device. The results of this study show that the LAPS cycle used did not induce unconsciousness rapidly and smoothly. Instead, the broilers were presumably conscious for a longer period than found in other studies (McKeegan et al., 2013; Mackie and McKeegan, 2016; Martin et al., 2016b; Gent et al., 2020). Therefore, it is questionable whether the LAPS system assessed in this study was at least equal to modern CAS systems such as those described in Santiago Rucinque et al. (2024). Currently, not only LAPS but also CAS systems provide enhanced animal welfare before stunning by avoiding the handling of live animals as compared with traditional stunning methods using an electrical water bath (Santiago Rucinque et al., 2024). In conclusion, the results of this study indicate that the use of LAPS for broiler stunning raises animal welfare concerns, which need to be investigated further. LAPS is authorized not only for stunning before slaughter but also for depopulation and other situations laid down in Commission Implementing Regulation (EU) 2018/723. In case of contagious diseases, especially zoonoses such as avian influenza, it is important to minimize the handling and transport of infected animals to protect humans and nearby stocks (Gerritzen et al., 2004). Currently, depopulation of broiler barns is usually done by floating the barn with gas (Gerritzen et al., 2004; Turner et al., 2012). This process can take up to 11 minutes (Turner et al., 2012) or even 30 minutes (Gerritzen et al., 2004) and is therefore critically discussed under welfare aspects. During LAPS, unconsciousness is reached after a maximum of 4:08 minutes, which is half as long as in other methods reported for depopulation of broiler barns, even though this time is very long from a welfare perspective. Further studies might therefore investigate using LAPS for depopulation of poultry houses, particularly after improving the LAPS decompression curve according to the ambient climatic condition and the different broiler genetics. In conclusion, LAPS-stunning in our study did not ensure a rapid and sustained onset of unconsciousness. The animals seemed to have a prolonged intermediate phase in which indicators of consciousness and unconsciousness occurred at the same time. LAPS in our study seemed to induce unconsciousness slower and less consistently as reported previously, which might negatively impact animal welfare because animals could potentially feel more pain. More studies, including behavioral and physiological parameters are needed, to finally assess the welfare consequences of LAPS-stunning.

## CRediT authorship contribution statement

**Felix Kuck:** Writing – original draft, Investigation, Data curation. **Jan Heck:** Writing – review & editing, Investigation. **Shana Bergmann:** Writing – review & editing, Supervision, Funding acquisition. **Paul Schmidt:** Visualization, Formal analysis. **Elke Rauch:** Writing – review & editing. **Helen Louton:** Writing – review & editing, Supervision. **Angela Schwarzer:** Writing – review & editing, Supervision, Project administration, Funding acquisition.

## Disclosures

The authors declare the following financial interests/personal relationships which may be considered as potential competing interests: Angela Schwarzer reports financial support was provided by Bavarian State Ministry of the Environment and Consumer Protection through the Bavarian Health and Food Safety Authority. If there are other authors, they declare that they have no known competing financial interests or personal relationships that could have appeared to influence the work reported in this paper.

## Appendix Supplementary materials

Image, application 1
