# Supplementary material for: Low atmospheric pressure stunning as a new method for stunning broilers in Germany under ethological aspects
Source: Poult Sci. 2025 Dec 10;105(2):106224. doi: 10.1016/j.psj.2025.106224 (PMC12800502; doi:10.1016/j.psj.2025.106224)
Supplement: Supplementary file 1 [file mmc1.docx]

# **Supplementary Material**

**Table** **S1.** Effects of live body weight in kilograms, body weight after bleeding in kilograms, age in days, and sex (female-to-male ratio) on the rate ratio (number of events) of the behaviors during the low atmospheric pressure stunning cycles.

| **Behavior** | **Factor influencing occurrence of the behavior** | | | |
| --- | --- | --- | --- | --- |
|  | **Live body weight (kg)**  **rate ratio**  **95% CI**  ***p*-value** | **Body weight after bleeding (kg)**  **rate ratio**  **95% CI**  ***p*-value** | **Age (days)**  **rate ratio**  **95% CI**  ***p*-value** | **Sex**  **(female-to-male ratio)**  **rate ratio**  **95% CI**  ***p*-value** |
| Standing | 1.37 [0.12, 14.63] *p* = 0.802 | 1.55 [0.13, 17.00] *p* = 0.728 | 0.83 [0.43, 1.49] *p* = 0.546 | 0.70 [0.35, 1.04] *p* < 0.001 |
| Sitting | 1.39 [0.31, .89] *p* = 0.662 | 1.41 [0.32, 6.13] *p* = 0.648 | 0.87 [0.59, 1.25] *p* = 0.467 | 0.39 [−0.35, 1.13] *p* = 0.302 |
| Ataxia | 1.39 [0.28, 6.72] *p* = 0.686 | 1.40 [0.27, 6.90] *p* = 0.686 | 1.04 [0.70, 1.51] *p* = 0.843 | −0.17 [−0.90, 0.57] *p* = 0.657 |
| Recovery of balance | 0.94 [0.28, 3.07] *p* = 0.924 | 0.89 [0.27, 2.96] *p* = 0.856 | 1.23 [0.93, 1.61] *p* = 0.144 | −0.09 [−1.07, 0.89] *p* = 0.862 |
| Alertness | 53.77 [4.93, 511.88] *p* < 0.001 | 57.13 [5.00, 567.66] *p* < 0.001 | 1.98 [1.09, 3.55] *p* = 0.022 | −0.58 [−1.09, -0.06] *p* = 0.030 |
| Drowsiness | 0.90 [0.30, 2.66] *p* = 0.853 | 0.89 [0.29, 2.66] *p* = 0.834 | 1.11 [0.86, 1.43] *p* = 0.421 | 1.11 [0.13, 2.09] *p* = 0.026 |
| Loss of posture | 1.00 [0.19, 5.13] *p* = 1.000 | 1.00 [0.18, 5.24] *p* = 1.000 | 1.00 [0.66, 1.47] *p* = 1.000 | 0.00 [−0.70, 0.70] *p* = 1.000 |
| Flip | 0.79 [0.07, 8.19] *p* = 0.844 | 0.81 [0.07, 8.66] *p* = 0.862 | 1.27 [0.72, 2.16] *p* = 0.381 | −0.02 [−0.51, 0.47] *p* = 0.931 |
| Lying | 0.89 [0.18, 4.29] *p* = 0.885 | 0.89 [0.18, 4.40] *p* = 0.889 | 0.96 [0.64, 1.40] *p* = 0.845 | 0.00 [−0.72, 0.73] *p* = 0.992 |
| Motionlessness | 1.81 [0.28, 10.85] *p* = 0.523 | 1.76 [0.27, 10.85] *p* = 0.549 | 0.90 [0.56, 1.40] *p* = 0.648 | −0.05 [−0.68, 0.58] *p* = 0.875 |
| Convulsion | 1.23 [0.64, 2.35] *p* = 0.528 | 1.21 [0.62, 2.32] *p* = 0.578 | 0.97 [0.82, 1.13] *p* = 0.688 | −1.97 [−3.82, −0.11] *p* = 0.038 |
| Wing flapping | 1.16 [0.39, 3.43] *p* = 0.790 | 1.21 [0.40, 3.62] *p* = 0.736 | 0.84 [0.63, 1.10] *p* = 0.225 | 0.69  [−0.32, 1.69]  *p* = 0.179 |
| Leg paddling | 1.25 [0.69, 2.25] *p* = 0.466 | 1.23 [0.67, 2.25] *p* = 0.492 | 1.07 [0.92, 1.23] *p* = 0.378 | −0.31 [−2.26, 1.64] *p* = 0.757 |
| Jumping | 0.16 [0.01, 3.72] *p* = 0.262 | 0.14 [0.01, 3.27] *p* = 0.225 | 0.86 [0.37, 1.79] *p* = 0.703 | 0.18 [−0.15, 0.51] *p* = 0.288 |
| Pecking movements | 3.66 [0.17, 63.51] *p* = 0.394 | 3.44 [0.15, 63.38] *p* = 0.425 | 0.30 [0.09, 0.78] *p* = 0.028 | 0.43 [0.17, 0.70] *p* = 0.002 |
| Normal breathing | 2.67 [1.11, 6.30] *p* = 0.027 | 2.63 [1.08, 6.29] *p* = 0.032 | 0.99 [0.79, 1.22] *p* = 0.913 | −1.21 [−2.57, 0.16] *p* = 0.084 |
| Mandibulation | 0.83 [0.31, 2.18] *p* = 0.703 | 0.78 [0.29, 2.09] *p* = 0.627 | 0.99 [0.78, 1.24] *p* = 0.905 | −0.38 [−1.60, 0.83] *p* = 0.536 |
| Deep inhalation | 1.60 [0.71, 3.57] *p* = 0.254 | 1.66 [0.73, 3.73] *p* = 0.227 | 0.77 [0.62, 0.95] *p* = 0.016 | 0.93 [−0.43, 2.30] *p* = 0.179 |
| Headshaking | 1.17 [0.39, 3.45] *p* = 0.780 | 1.20 [0.39, 3.59] *p* = 0.749 | 0.89 [0.68 1.17] *p* = 0.418 | −0.58 [−1.68, 0.52] *p* = 0.300 |
| Open bill (beak) breathing | 1.15 [0.05, 22.90] *p* = 0.929 | 0.88 [0.04, 18.85] *p* = 0.939 | 0.86 [0.37, 1.79] *p* = 0.703 | −0.71 [−1.20, −0.21] *p* = 0.005 |
| Loss of jaw tension | 2.95 [1.22, 6.99] *p* = 0.015 | 2.86 [1.17, 6.88] *p* = 0.020 | 1.21 [0.98, 1.48] *p* = 0.077 | −2.56 [−3.99, −1.13] *p* < 0.001 |
| Eyes open | 1.15 [0.58, 2.27] *p* = 0.686 | 1.15 [0.57, 2.29] *p* = 0.694 | 1.07 [0.91, 1.26] *p* = 0.399 | 0.80 [−0.86, 2.45] *p* = 0.344 |
| Eyes half-open | 1.34 [0.62, 2.89] *p* = 0.456 | 1.32 [0.60, 2.87] *p* = 0.488 | 1.00 [0.83, 1.20] *p* = 1.000 | 0.07 [−1.42, 1.55] *p* = 0.931 |
| Eyes closed | 0.56 [0.27, 1.14] *p* = 0.109 | 0.54 [0.26, 1.12] *p* = 0.101 | 1.00 [0.84, 1.18] *p* = 1.000 | 1.33 [−0.24, 2.90] *p* = 0.096 |
| Blinking | 0.70 [0.48, 1.01] *p* = 0.054 | 0.70 [0.48, 1.01] *p* = 0.057 | 0.93 [0.85, 1.02] *p* = 0.114 | 0.06 [−3.09, 3.21] *p* = 0.971 |
| Vocalizing | 0.47 [0.04, 5.90] *p* = 0.568 | 0.41 [0.03, 5.25] *p* = 0.496 | 1.55 [0.87, 2.69] *p* = 0.122 | 0.53 [0.17, 0.88] *p* = 0.004 |
| Ruffled plumage | 3.86 [0.63, 21.85] *p* = 0.137 | 4.12 [0.66, 23.96] *p* = 0.124 | 0.95 [0.59, 1.47] *p* = 0.819 | −0.18 [−0.82, 0.47] *p* = 0.590 |
| Defecation | 1.46 [0.15, 12.89] *p* = 0.739 | 1.43 [0.14, 13.06] *p* = 0.756 | 0.85 [0.47, 1.46] *p* = 0.580 | −0.19 [−0.73, 0.35] *p* = 0.495 |

CI = confidence interval

**Table** **S2.** Effects of live body weight in kilograms, body weight after bleeding in kilograms, and age in days expressed as the slope (duration) in seconds and of sex (female-to-male ratio) expressed as the difference in means in seconds on the behaviors during the low atmospheric pressure stunning cycles.

| **Behavior** | **Factor influencing occurrence of the behavior** | | | |
| --- | --- | --- | --- | --- |
|  | **Live body weight (kg)**  **slope (seconds)**  **95% CI**  ***p*-value** | **Body weight after bleeding (kg)**  **slope (seconds)**  **95% CI**  ***p*-value** | **Age (days)**  **slope (seconds)**  **95% CI**  ***p*-value** | **Sex (female-to-male ratio)**  **difference in means (seconds)**  **95% CI**  ***p*-value** |
| Standing | 22.24 [−124.65, 16.12] *p* = 0.695 | 24.79 [−126.49, 176.0]7 *p* = 0.671 | 8.87 [−30.16, 47.90] *p* = 0.504 | X |
| Sitting | −0.57 [−46.38, 45.24] *p* = 0.980 | −2.72 [−49.14, 43.70] *p* = 0.906 | 6.19 [−4.82, 17.21] *p* = 0.260 | −20.80 [−38.8, −2.80]  *p* = 0.025 |
| Ataxia | 4.05 [−13.04, 21.15] *p* = 0.633 | 4.68 [−12.60, 21.95] *p* = 0.585 | 3.13 [−0.94, 7.19] *p* = 0.127 | 0.43 [−6.82, 7.68)] *p* = 0.905 |
| Recovery of balance | 0.60 [−2.21, 3.41] *p* = 0.651 | 0.56 [−2.36, 3.48] *p* = 0.684 | 0.20 [−0.47, 0.88] *p* = 0.522 | 0.17 [−0.82, 1.15] *p* = 0.721 |
| Alertness | 1.73 [−53.27, 56.73] *p* = 0.941 | 0.02 [−55.24, 55.29] *p* = 0.999 | −1.01 [−17.65, 15.63] *p* = 0.881 | −14.94 [−38.46, 8.59] *p* = 0.182 |
| Drowsiness | 10.88 [−7.51, 29.27] *p* = 0.237 | 11.46 [−7.23, 30.16] *p* = 0.222 | 3.35 [−0.42, 7.12] *p* = 0.078 | 0.69 [−6.21, 7.58] *p* = 0.842 |
| Loss of posture | −2.83 [−18.03, 12.37] *p* = 0.707 | −2.22 [−17.62, 13.19] *p* = 0.772 | −3.71 [−7.14, −0.27] *p* = 0.035 | 8.53 [2.87, 14.19] *p* = 0.004 |
| Flip | 0.00 [0.00, .00] *p* = 0.855 | 0.00 [0.00, 0.00] *p* = 0.901 | 0.00 [0.00, 0.00] *p* = 0.667 | 0.00 [0.00, 0.00] *p* = 0.667 |
| Lying | 28.15 [−48.57, 104.88] *p* = 0.458 | 26.90 [−50.68, 104.48] *p* = 0.483 | 3.99 [−14.23, 22.22] *p* = 0.658 | −7.39 [−38.94, 24.16] *p* = 0.637 |
| Motionlessness | −20.12 [−70.64, 30.39] *p* = 0.420 | −19.99 [−71.06, 31.08] *p* = 0.428 | −7.00 [−18.55, 4.56] *p* = 0.224 | 7.99 [−13.7, 29.68] *p* = 0.456 |
| Convulsion | 0.59 [−6.50, 7.69] *p* = 0.866 | 0.60 [−6.58, 7.78] *p* = 0.866 | 0.66 [−1.05, 2.37] *p* = 0.436 | 1.21 [−1.72, 4.14] *p* = 0.407 |
| Wing flapping | −0.07 [−5.04, 4.90] *p* = 0.978 | −0.38 [−5.29, 4.53] *p* = 0.874 | 0.07 [−1.12, 1.26] *p* = 0.908 | −0.90 [−2.58, 0.78] *p* = 0.274 |
| Leg paddling | 0.95 [−1.92, 3.83] *p* = 0.506 | 0.89 [−2.00, 3.78] *p* = 0.535 | 0.10 [−0.60, 0.79] *p* = 0.774 | −0.07 [−1.25, 1.11] *p* = 0.904 |
| Jumping | X | X | X | X |
| Pecking movements | X | X | X | X |
| Normal breathing | −79.81 [−144.41, −15.21] *p* = 0.017 | −79.14 [−144.92, −13.36] *p* = 0.020 | −1.72 [−18.98, 15.55] *p* = 0.841 | 34.20 [8.03, 60.38)] *p* = 0.012 |
| Mandibulation | −0.42 [−10.65, 9.81] *p* = 0.929 | −0.41 [−11.16, 10.35] *p* = 0.934 | 0.49 [−1.50, 2.49] *p* = 0.598 | −0.04 [−4.05, 3.97] *p* = 0.981 |
| Deep inhalation | 9.88 [−8.23, 27.99] *p* = 0.224 | 10.29 [−8.60, 29.18] *p* = 0.221 | 1.46 [−1.11, 4.02] *p* = 0.228 | −2.48 [−13.71, 8.74)] *p* = 0.396 |
| Headshaking | −2.34 [−10.41, 5.72] *p* = 0.522 | −2.28 [−10.38, 5.83] *p* = 0.536 | −0.13 [−2.19, 1.94] *p* = 0.892 | −0.38 [−3.76, 3.00] *p* = 0.808 |
| Open bill (beak) breathing | −7.81 [−23.02, 7.39] *p* = 0.314 | −7.94 [−13.29, −2.58] *p* = 0.009 | −3.50 [−5.93, −1.07] *p* = 0.020 | 3.50 [1.14, 5.86] *p* = 0.009 |
| Loss of jaw tension | 9.33 [−7.71, 26.37] *p* = 0.275 | 9.11 [−7.94, 26.16] *p* = 0.286 | 1.62 [−1.87, 5.12] *p* = 0.347 | 1.24 [−4.45, 6.94] *p* = 0.654 |
| Eyes open | −4.28 [−36.63, 28.07] *p* = 0.792 | −5.22 [−37.73, 27.29] *p* = 0.748 | −1.20 [−7.76, 5.36] *p* = 0.708 | −3.53 [−14.14, 7.07] *p* = 0.501 |
| Eyes half-open | −2.90 [−21.30, 15.50] *p* = 0.751 | −2.50 [−20.87, 15.86] *p* = 0.783 | −0.62 [−4.61, 3.38] *p* = 0.749 | −3.49 [−9.19, 2.21] *p* = 0.217 |
| Eyes closed | 3.06 [−9.50, 15.62] *p* = 0.623 | 3.29 [−9.40, 15.97] *p* = 0.601 | −0.32 [−3.47, 2.83] *p* = 0.838 | −1.66 [−7.18, 3.87] *p* = 0.545 |
| Blinking | −1.49 [−3.03, 0.05] *p* = 0.058 | −1.52 [−3.07, 0.04] *p* = 0.056 | −0.31 [−0.70, 0.09] *p* = 0.128 | 0.13 [−0.56, 0.81] *p* = 0.703 |
| Vocalizing | 4.52 [−3,081.96, 3,091.01] *p* = 0.564 | 4.90 [−4,621.21, 4,631.02] *p* = 0.558 | 0.60 [−10.57, 11.77] *p* = 0.564 | X |
| Ruffled plumage | −9.36 [−44.43, 25.71] *p* = 0.585 | −9.25 [−44.59, 26.08] *p* = 0.592 | 3.78 [−4.42, 11.97] *p* = 0.350 | −0.33 [−16.62, 15.96] *p* = 0.966 |
| Defecation | X | X | X | X |

CI = confidence interval; X = no calculation possible owing to lack of data
